# Reconsidering the associations between self-reported alcohol use disorder and mental health problems in the light of co-occurring addictions in young Swiss men

**DOI:** 10.1371/journal.pone.0222806

**Published:** 2019-09-30

**Authors:** Simon Marmet, Joseph Studer, Mélissa Lemoine, Véronique S. Grazioli, Nicolas Bertholet, Gerhard Gmel

**Affiliations:** 1 Addiction Medicine, Lausanne University Hospital and University of Lausanne, Lausanne, Switzerland; 2 Addiction Switzerland, Lausanne, Switzerland; 3 Centre for Addiction and Mental Health, Toronto, Canada; 4 University of the West of England, Frenchay Campus, Bristol, United Kingdom; East Tennessee State University, UNITED STATES

## Abstract

**Background:**

Alcohol use disorder (AUD) is known to co-occur with other addictions, as well as with mental health problems. However, the effects of other addictions co-occurring with AUD on mental health problems were rarely studied and not considering them may bias estimates of the association between AUD and mental health problems. This study investigated which role co-occurring addictions play for the cross-sectional associations between self-reported AUD and mental health problems.

**Method:**

Participants were 5516 young Swiss men (73.0% of those that gave written informed consent) who completed a self-report questionnaire. Using short screening questionnaires, we assessed three substance use disorders (alcohol, cannabis and tobacco), seven behavioural addictions (internet, gaming, smartphone, internet sex, gambling, work, exercise) and four mental health problems (major depression, bipolar disorder, attention deficit hyperactivity disorder (ADHD) and social anxiety disorder). Differences in the proportions of mental health problems were tested using logistic regressions between (1) participants with no AUD and AUD, (2) participants with no AUD and AUD alone and (3) participants with no AUD and AUD plus at least one co-occurring addiction.

**Results:**

Overall, (1) participants with AUD had higher proportions of major depression (Odds ratio (OR [95% confidence interval]) = 3.51 [2.73, 4.52]; ADHD (OR = 3.12 [2.41, 4.03]); bipolar disorder (OR = 4.94 [3.38, 7.21]) and social anxiety (OR = 2.21 [1.79, 2.73])) compared to participants with no AUD. Considering only participants with AUD alone compared to participants with no AUD (2), differences in proportions were no longer significant for major depression (OR = 0.83 [0.42, 1.64]), bipolar disorder (OR = 1.69 [0.67, 4.22]), social anxiety (OR = 1.15 [0.77, 1.73]) and ADHD (OR = 1.65 [1.00, 2.72]) compared to participants with no AUD. In contrast, (3) proportions of mental health problems were considerably higher for participants with AUD plus at least one other addiction when compared to participants with no AUD, with OR’s ranging from 2.90 [2.27, 3.70] for social anxiety, 4.03 [3.02, 5.38] for ADHD, 5.29 [4.02, 6.97] for major depression to 6.64 [4.44, 9.94] for bipolar disorder.

**Conclusions:**

AUD was associated with all four measured mental health problems. However, these associations were mainly due to the high proportions of these mental health problems in participants with AUD plus at least one co-occurring addiction and only to a lesser degree due to participants with AUD alone (i.e. without any other co-occurring addictions). Hence, estimates of the association between AUD and mental health problems that do not consider other addictions may be biased (i.e. overestimated). These findings imply that considering addictions co-occurring with AUD, including behavioural addictions, is important when investigating associations between AUD and mental health problems, and for the treatment of AUD and co-morbid disorders.

## 1. Introduction

Alcohol use is widespread among young men in Switzerland [1] and a considerable proportion shows symptoms of alcohol use disorder (AUD) [2]. AUD is known to co-occur with other substance use disorders (SUDs) and non-substance related addictive behaviours (i.e. behavioural addictions (BAs)), however, there are few estimates of the prevalence of these co-occurrences [3–5] and they vary widely. Mental health problems (MHPs) are also known to be associated with AUD and other addictions [6], but, likewise, few studies have investigated the links between MHPs and the co-occurrence of different SUDs and BAs. This article investigates the co-occurrence of two SUDs and seven BAs with AUD, as well as the effects of these co-occurrences on the associations between AUD and four MHPs, namely major depression (MD), bipolar disorder, attention deficit hyperactivity disorder (ADHD) and social anxiety disorder, which have been found to be associated with AUD [7–10]. Among the non-substance related addictive disorders, only gambling disorder is currently included as a disorder in the DSM-5, and internet gaming is under consideration for inclusion pending further research [11]. There is still an ongoing debate for several BAs about how they should be measured and classified [12]. Also, there are different terms in use for describing addictive disorders, such as use disorders, problematic use or compulsive use. While we acknowledge these heterogeneities in definition and terminology, the term “addiction” is used in this paper to refer to SUDs and BAs for the ease of reading.

Not only do different SUDs and BAs tend to co-occur [3], but it has also been found that the co-occurrence of MHPs and SUDs is common, particularly in younger people [6]. Specifically for alcohol, a review found that the presence of AUD approximately doubled the risk for major depression and vice-versa [7]. For ADHD, a meta-analysis found that about 23% of participants with SUDs (19.9% to 28.6% for AUD) also showed signs of ADHD [8]. Another review found that AUD affected more than a third of patients with bipolar disorder [9]. Social anxiety disorder was found to increase the odds of alcohol dependence by 2.8 (by 1.2 for alcohol abuse), and the combination of social anxiety disorder and AUD was associated with increased prevalence rates for other SUDs and pathological gambling [10]. Generally, individuals with SUDs and other MHPs tend to have a more severe course of illness, suffer from more severe health and social consequences, complications and worse outcomes in treatment [6].

Concerning the association of multiple SUDs with MHPs, a study using the USA’s Veterans Health Administration register among 472,642 veterans with at least one SUD (not including tobacco) found that 26.8% of those had at least two SUDs. Having two or more SUDs was associated with more medical and mental health disorders, particularly bipolar and depressive disorders [13]. Associations were even stronger for patients with more than two co-occurring SUDs. In the same sample, patients with both AUD and tobacco use disorders were shown to have more problems than those with either condition alone [14]. A study in 125 male patients in substance use treatment also found that polysubstance dependence was associated with a higher risk for anxiety disorder, bipolar disorder as well as axis II disorders compared to those with AUD only, respectively no SUD at all [15]. A study based on representative sample of the United States’ population found that in alcohol dependent individuals a pattern of polysubstance use including, tobacco, cannabis and illicit drugs was associated with higher psychopathological comorbidity, among them major depression, social phobia and borderline personality disorder, compared to those with alcohol use only [16]. Multiple studies also reported associations between polysubstance use and psychiatric comorbidities[17–20], respectively psychological distress [21]. A study in a general population sample also found that co-occurring tobacco use disorder in participants with AUD increases the risk of a broad spectrum of MHPs, including other addictive disorders [22].

### 1.1 Aims

Better knowledge of the interplay between SUDs, BAs and MHPs could be of considerable interest for research and treatment purposes. Although there is evidence for the co-occurrence of AUD and other SUDs being associated with MHPs, to the best of our knowledge, no studies have investigated the associations between a broad range of addictions, including BAs, co-occurring with AUD and MHPs. Also, if co-occurring addictions were in part responsible for the association between AUD and MHPs, the association between AUD and MHPs may have been biased (i.e. overestimated) in most past studies not taking into account addictions co-occurring with AUD. The present study aimed to investigate these questions by taking advantage of the data from the Cohort Study on Substance Use Risk Factors (C-SURF), which covers several SUDs, BAs and MHPs and features a large sample size. More specifically, the present study investigated a) the co-occurrence of other addictions with AUD, and b) how the associations between AUD and MHPs change when other addictions co-occurring with AUD are taken into account.

## 2. Method

### 2.1 Sample

The sample came from the C-SURF study (www.c-surf.ch), a cohort study designed to examine substance use patterns and related factors in young Swiss men (for an overview, see [23, 24]). Enrolment for the baseline assessment took place between August 2010 and November 2011 during the recruitment procedure for military service (mandatory for all young Swiss men) at three of the six Swiss military recruitment centres, located in Lausanne, Windisch and Mels, covering 21 of 26 Swiss cantons. As these recruitment procedures are mandatory for all young Swiss men, there is no a priori selectivity in the sampling strategy. Out of 13’237 young men that have been asked to participate in the study, 7556 gave their written consent to participate in the study after having been informed about the aims of the study, and the possibility to withdraw from the study at any time. Differences between consenters and non-consenters to the study were only small, and did not introduce bias in this sample [25]. Of these, 5987 returned the baseline questionnaire and 5516 (of which 391 did not complete the baseline questionnaire) returned the second follow-up questionnaire (wave 3) between April 2016 and March 2018. Among consenters, differences between respondents and nonrespondents were generally small regarding substance use and misuse, indicating that the risk of nonresponse bias was small [24]. Mean ages were 19.97 (SD = 1.22) years old at baseline and 25.47 (SD = 1.26) at wave 3 (2^nd^ follow-up). Study procedures were independent of the military and participants filled out the questionnaire at home either on paper or online. Participants received vouchers (50 Swiss francs for the third questionnaire, this corresponds to the price of about two to three cinema tickets) as a thank-you for their efforts. The present study uses data from wave 3 only, as most behavioural addictions were measured only in this wave. The research protocol was approved by the Human Research Ethics Committee of the Canton Vaud (Protocol No. 15/07).

### 2.2 Measures

#### 2.2.1 Mental health

*Major depression* in the last two weeks was measured using the Major Depression Inventory (WHO-MDI) [26] consisting of 12 statements measuring 10 criteria with 6-point Likert-type answers ranging from “never” (0) to “always” (5). Two criteria were assessed with two statements, and only the highest value of the two statements was used for building the sum score. The sum of the 10 criteria was built and a cut-off of 21 points out of 50 possible was used to represent “at least mild major depression” [27]. Cronbach’s Alpha for this scale was .90 in this sample, indicating acceptable internal consistency (alpha >.70 [28–30])

*Bipolar disorder* (lifetime) was measured using the Mood Disorder Questionnaire (MDQ) [31, 32], a screener for bipolar spectrum disorder. Participants fulfilling the criteria had to report at least 7 of the 13 symptoms, and some symptoms had to occur in the same time frame and they had to cause at least moderate problems. Cronbach’s Alpha for this scale was .82 in this sample.

*Adult attention deficit hyperactivity disorder (ADHD)* in the past 12 months was measured using the six-item screener version of the Adult ADHD Self-Report Scale (ASRS-v1.1; [33]) developed by the World Health Organization (WHO). Response options were on a 5-point Likert-type scale ranging from “never” to “very often”. Four or more items with at least “sometimes” (first 3 items), respectively “often” (last 3 items) were defined as the threshold for ADHD. Cronbach’s Alpha for this scale was .78 in this sample.

*Social anxiety disorder* during the past week was measured with the Clinically Useful Social Anxiety Disorder Outcome Scale (CUSADOS; [34]) consisting of 12 statements with 5-point Likert options ranging from “almost never true” to “almost always true”. A cut-off score of 16 was used for identifying participants with social anxiety disorder. Cronbach’s Alpha for this scale was .93 in this sample.

#### 2.2.2 Substance use disorder and behavioural addiction scales

*Alcohol use disorder* (last 12 months) was measured using 12 items for the 11 DSM-5 criteria [11, 35, 36] in a yes/no format. The DSM-5 moderate (4+ criteria) cut-off was chosen for AUD because mild (2+ criteria) AUD is very frequent in young men (almost a third in this sample; [37]) and would therefore inflate occurrences of AUD as well as co-occurrence with other addictions. Cronbach’s Alpha for this scale was .70 in this sample.

*Cannabis use disorder* (last 12 months) was measured using the revised version of the Cannabis Use Disorder Identification Test (CUDIT-R; [38], based on [39]). The test consists of ten items, and a cut-off of 8 out of 40 points was used. Cronbach’s Alpha for this scale was .85 in this sample.

*Tobacco dependence* (last 12 months) was assessed using the Fagerström Test for Nicotine Dependence (six items; FTND; [40, 41]), with a cut-off of 3 out of 10 possible points. Cronbach’s Alpha for this scale was .71 in this sample.

*Internet addiction* (current without specified time frame) was measured using the Compulsive Internet Use Scale (CIUS; fourteen 5-point items), with a cut-off of 28 points [42, 43]. Cronbach’s Alpha for this scale was .92 in this sample.

*Gaming disorder* (last 6 months) was measured using the Game Addiction Scale, with seven items ranging from “never” to “very often”, and participants who responded to at least four items with at least “sometimes” were defined as presenting a gaming disorder [44]. Cronbach’s Alpha for this scale was .85 in this sample.

*Smartphone addiction* (current without specified time frame) was measured with the Smartphone Addiction Scale (ten 5-point items), with a cut-off of 31 points [45, 46]. Cronbach’s Alpha for this scale was .88 in this sample.

*Internet sex addiction* (last 12 months) was measured using the online sexual compulsivity subscale from the Internet Sex Screening Test (ISST; [47]), and participants who agreed to at least three out of six items (corresponding to “risky” for the full scale [48]) were defined as presenting with an internet sex addiction. Cronbach’s Alpha for this scale was .64 in this sample.

*Gambling disorder* (last 12 months) was measured using nine yes/no items representing the DSM-5 criteria (DSM-5; [11], translated from [49]) with a cut-off of four *yes* answers equating to a mild gambling disorder. Cronbach’s Alpha for this scale was .85 in this sample.

*Work addiction* (last 12 months) was measured using the Bergen Work addiction scale [50], with seven 5-point Likert-type items ranging from “never” to “always“. Participants answering at least four items with at least “often” were defined as presenting a work addiction. Cronbach’s Alpha for this scale was .78 in this sample.

*Exercise addiction* (current without specified time frame) was assessed using the Exercise Addiction Inventory [51] with six 5-point items and a cut-off of 24 points. Cronbach’s Alpha for this scale was .89 in this sample.

### 2.3 Statistical analysis

All analysis were done in SPSS 25. Multiple imputation with fully conditional specification for 25 imputed datasets was used for items of the addiction and MHP scales. Internal consistency of the scales was estimated using the Cronbach’s Alpha statistic. All scales except the internet sex addiction scale (alpha = .64) were in the range of what is often considered as being an acceptable internal consistency (alpha >.70; [28–30].

Associations between AUD, co-occurring addictions and MHPs were tested in three steps: step 1 was the “common” comparison used by most studies looking at the link between AUD and mental health, i.e. testing for differences in the proportions of MHPs between participants without AUD (reference group) and with AUD. In step 2, to test whether the association between AUD and MHPs is mainly due to AUD alone or mainly due to other addictions co-occurring with AUD, participants with AUD were split into one group with AUD alone and another with AUD plus at least one other addiction. Both groups were again compared with the reference group without AUD. However, the reference group in steps 1 and 2 contained participants with addictions other than AUD, which may themselves be associated with MHPs. In step 3, therefore, participants with any of the nine other addictions measured in addition to AUD were removed from the reference group, creating a reference group of participants with none of the ten addictions measured. This *no-addiction* reference group was then compared to the AUD alone group, the group with AUD plus at least one other addiction, and additionally to the participants with one, respectively two or more other addictions.

To examine the association between MHPs and specific addictions co-occurring with AUD, the associations of MHPs with each of the nine co-occurring addictions were tested in participants with AUD separately (with only one addiction as the independent variable plus sociodemographics; Model 1) and simultaneously (adjusted associations with all nine addictions as independent variables plus sociodemographics; Model 2). Given that addictions are correlated, multicollinearity diagnostics were performed for Model 2 using the linear regression function in SPSS as recommended in [52]. Variance inflation factors were below 1.3 for all addictions, indicating that there was no problem with multicollinearity [52].

The associations between mental health problems and subgroups of participants with AUD were analysed using logistic regression models. In all models, the dependent variable were the presence of each MHP. The different subgroups of participants (i.e. participants with no AUD; only AUD, and AUD plus other addictions) were entered as categorical predictor variable into the regression models.

All regressions were adjusted for sociodemographic variables: age, linguistic region (French vs. German) and highest level of education (secondary school, apprenticeship or higher education). Results are reported as Odds ratios (OR) of having an MHP versus not having an MHP with 95% confidence intervals. Chi-square tests were conducted to test whether addictions are more frequent in participants with AUD compared to those without AUD.

## 3. Results

Table 1 shows the descriptive statistics for the sample. Almost half (45.2%) of participants reported having at least one of the ten addictions, with 8.9% *(n =* 488) having at least moderate (four or more criteria) AUD. Of those with at least a moderate AUD, slightly more than a third reported only having AUD, about as many reported one co-occurring addiction, and the remainder reported two or more addictions in addition to AUD (Table 2). The most frequent co-occurring addictions with AUD were tobacco (27.7%) and cannabis (21.1%). All measured addictions, except exercise, were significantly more frequent (*p* < .01; tests not shown) in participants with AUD than in those without.

**Table 1:** Descriptive statistics and proportions of mental health problems in the total sample (n = 5516).

		n	mean/%
	Age (mean/SD)	5516	25.47 (1.26)
Highest education			
	Primary schooling	262	4.7%
	Apprenticeship	1966	35.6%
	Higher education	3288	59.6%
Linguistic region			
	French-speaking	3179	57.6%
	German-speaking	2337	42.4%
Mental health problems			
	Mild major depression	435	7.9%
	ADHD	428	7.8%
	Bipolar disorder	144	2.6%
	Social anxiety disorder	923	16.7%
AUD and other addictions			
	Any addiction	2494	45.2%
	AUD (at least moderate)	488	8.9%

Note: AUD = alcohol use disorder. ADHD = attention deficit hyperactivity disorder.

**Table 2:** Co-occurrence of substance use disorders and behavioural addictions with alcohol use disorder (at least moderate; n = 488).

	Substance use disorders			Behavioural addictions						
	Alcohol	Cannabis	Tobacco	Internet	Gaming	Smart- phone	Internet sex	Gambling	Work	Exercise
AUD alone (n)	169	0	0	0	0	0	0	0	0	0
AUD + 1 other (n)	170	39	51	4	9	22	23	2	15	5
AUD + 2 or more others (n)	150	64	84	50	44	68	50	21	37	6
Total (n)	488	103	135	54	53	89	73	23	52	11
% of participants with AUD reporting this addiction	100.0%	21.1%	27.7%	11.1%	10.9%	18.3%	14.9%	4.8%	10.6%	2.3%
Proportion (%) of addiction in total sample	8.9%	8.0%	16.8%	4.7%	7.0%	8.1%	7.1%	1.5%	8.1%	2.9%

Note: AUD = alcohol use disorder.

All four MHPs were significantly more frequent in participants with AUD than without AUD (Table 3, step 1). Among participants with AUD alone (Table 3, step 2a), proportions of MHPs were not significantly higher than among participants without AUD (but possibly *with* other addictions). Conversely, proportions of MHPs were higher among participants with AUD plus at least one other co-occurring addiction than among participants without AUD (Table 3, step 2a). Compared to participants with AUD alone (step 2b), those with AUD plus at least one other co-occurring addiction were also more likely to report MHPs, with ORs ranging from 2.45 [1.40, 4.28] for ADHD to 6.38 [3.11, 13.09] for MD. Additionally, there was a steep increase in the proportions of MHPs with the number (one to three-or-more) of co-occurring addictions (step 2c).

**Table 3:** Proportions and results of logistic regressions for major depression, ADHD, bipolar disorder and social anxiety disorder by DSM-5 alcohol use disorder (AUD) status and other co-occurring addictions (n = 5516).

		Major depression		ADHD		Bipolar disorder		Social anxiety disorder	
**Step 1: no AUD (ref.) vs AUD**	n	%	OR [95% CI]	%	OR [95% CI]	%	OR [95% CI]	%	OR [95% CI]
No AUD (ref.)	5028	6.7%	ref.	6.7%	ref.	2.0%	ref.	15.6%	ref.
AUD	488	19.8%	**3.51 [2.73, 4.52]**	18.3%	**3.12 [2.41, 4.03]**	8.7%	**4.94 [3.38, 7.21]**	28.8%	**2.21 [1.79, 2.73]**
**Step 2a: no AUD (ref.) vs AUD with other co-occurring addictions**									
No AUD (ref.)	5028	6.7%	ref.	6.7%	ref.	2.0%	ref.	15.6%	ref.
AUD alone	169	5.4%	0.83 [0.42, 1.64]	10.7%	1.65 [1.00, 2.72]	3.0%	1.69 [0.67, 4.22]	17.3%	1.15 [0.77, 1.73]
AUD + 1 or more other addictions	320	27.4%	**5.29 [4.02, 6.97]**	22.2%	**4.03 [3.02, 5.38]**	11.7%	**6.64 [4.44, 9.94]**	34.8%	**2.90 [2.27, 3.70]**
**Step 2b: AUD alone (ref.) vs AUD with other co-occurring addictions**									
AUD alone	169	5.4%	ref.	10.7%	ref.	3.0%	ref.	17.3%	ref.
AUD + 1 or more other addictions	320	27.4%	**6.38 [3.11, 13.09]**	22.2%	**2.45 [1.40, 4.28]**	11.7%	**3.94 [1.51, 10.30]**	34.8%	**2.52 [1.59, 4.00]**
**Step 2c: AUD alone (ref.) vs AUD with a number of other co-occurring addictions**									
AUD alone	169	5.4%	ref.	10.7%	ref.	3.0%	ref.	17.3%	ref.
AUD + 1 other addiction	170	16.3%	**3.38 [1.54, 7.46]**	19.1%	**2.06 [1.10, 3.87]**	7.6%	2.52 [0.87, 7.31]	25.7%	1.64 [0.97, 2.79]
AUD + 2 other addictions	72	27.6%	**6.50 [2.77, 15.29]**	18.8%	2.00 [0.92, 4.37]	11.6%	**3.81 [1.17, 12.40]**	32.5%	**2.28 [1.20, 4.34]**
AUD + 3 or more other addictions	78	51.1%	**16.78 [7.46, 37.78]**	32.2%	**3.85 [1.93, 7.69]**	20.7%	**7.41 [2.57, 21.33]**	56.7%	**6.11 [3.33, 11.21]**
**Step 3: no addiction (ref.) vs AUD alone and AUD with other co-occurring addictions and multiple other addictions (total number of addictions)**									
No addiction (ref.)	3022	3.3%	ref.	3.8%	ref.	0.6%	ref.	10.6%	ref.
AUD alone	169	5.4%	1.71 [0.85, 3.45]	10.7%	**2.97 [1.76, 5.02]**	3.0%	**5.39 [1.97, 14.76]**	17.3%	**1.77 [1.17, 2.69]**
AUD + 1 or more other addictions	320	27.4%	**11.03 [8.00, 15.21]**	22.2%	**7.37 [5.32, 10.21]**	11.7%	**21.51 [12.07, 38.35]**	34.8%	**4.51 [3.48, 5.84]**
No AUD, 1 other addiction	1326	7.5%	**2.33 [1.74, 3.11]**	8.4%	**2.35 [1.79, 3.08]**	2.7%	**4.22 [2.38, 7.48]**	16.4%	**1.64 [1.36, 1.98]**
No AUD, 2 or more other addictions	679	20.5%	**7.23 [5.48, 9.54]**	16.4%	**4.98 [3.77, 6.59]**	7.0%	**11.03 [6.32, 19.22]**	36.1%	**4.74 [3.88, 5.78]**

Notes: AUD = alcohol use disorder. ADHD = attention deficit hyperactivity disorder. Ref. = reference category for logistic regression. CI = confidence interval. ORs in bold are significant at p < .05. Adjusted for age, linguistic region and highest level of education.

The model in step 2a did not take into account that the reference group contained participants with addictions other than AUD. When instead the participants with none of the addictions measured were used as the reference group (Table 3, step 3), ORs for MHPs were higher for participants with AUD alone than in step 2a. Proportions of MHPs in step 3 were considerably higher among participants with AUD plus at least one co-occurring addiction than among participants showing *no addiction*, with ORs ranging from 4.51 [3.48, 5.84] for social anxiety disorder to 21.51 [12.07, 38.35] for bipolar disorder.

Overall, a combination of AUD with any other addiction was associated with higher proportions of MHPs (Tables 4 and 5, Model 1), except with gambling for ADHD and exercise for bipolar disorder (no OR was calculated here because there was no complete case with bipolar disorder and exercise addiction), but not all of these associations were statistically significant, mainly due to the small number of cases for some comparisons. When adjusting for the presence of the other eight addictions, these associations were considerably weakened, indicating some confounding by a third (or additional) addiction. Nevertheless, several associations remained significant even after adjustment for the presence of additional addictions (Tables 4 and 5, Model 2), but only internet and work addiction still showed significant associations across all four MHPs.

**Table 4:** Proportions of mental health problems among participants with alcohol use disorder and co-occurring addictions, adjusted for sociodemographic variables (Model 1, 2) and other addictions (Model 2; n = 488).

		Major depression			ADHD		
AUD combined with addiction to…	n	%	Model 1 OR [95% CI]	Model 2 OR [95% CI]	%	Model 1 OR [95% CI]	Model 2 OR [95% CI]
Alcohol (total)	488	19.8%			18.3%		
Cannabis	103	31.2%	**2.10 [1.25, 3.52]**	1.55 [0.84, 2.85]	24.3%	**1.58 [0.92, 2.72]**	1.20 [0.66, 2.19]
Tobacco	135	32.3%	**2.66 [1.63, 4.32]**	**2.34 [1.36, 4.05]**	21.5%	1.49 [0.89, 2.49]	1.45 [0.83, 2.53]
Internet	54	46.4%	**4.34 [2.35, 7.99]**	**2.54 [1.22, 5.26]**	39.0%	**3.10 [1.66, 5.76]**	**2.73 [1.34, 5.58]**
Gaming	53	47.7%	**4.75 [2.57, 8.78]**	**2.86 [1.42, 5.79]**	28.5%	**2.13 [1.10, 4.14]**	1.70 [0.82, 3.49]
Smartphone	89	30.4%	**2.26 [1.32, 3.87]**	1.40 [0.73, 2.71]	19.2%	1.15 [0.63, 2.09]	0.75 [0.38, 1.51]
Internet sex	73	26.0%	1.51 [0.83, 2.77]	1.24 [0.61, 2.51]	30.2%	**2.01 [1.12, 3.61]**	**1.92 [1.02, 3.59]**
Gambling	23	47.4%	**3.64 [1.51, 8.79]**	1.87 [0.66, 5.32]	12.9%	0.68 [0.19, 2.42]	0.38 [0.10, 1.45]
Work	52	50.0%	**5.48 [2.90, 10.34]**	**5.10 [2.57, 10.12**]	35.0%	**2.46 [1.29, 4.70]**	**2.18 [1.10, 4.33]**
Exercise	11	26.7%	1.65 [0.43, 6.38]	1.79 [0.38, 8.50]	28.0%	2.02 [0.51, 8.03]	1.98 [0.45, 8.67]

Notes: AUD = alcohol use disorder. ADHD = attention deficit hyperactivity disorder. ORs in bold are significant at p < .05. CI = 95% confidence interval. Adjusted for age, linguistic region and highest level of education (Model 1, 2). Model 2 is additionally adjusted for all other addictions (e.g. the regression for cannabis is adjusted by the eight addictions other than AUD and cannabis).

**Table 5:** Proportions of mental health problems among participants with alcohol use disorder and co-occurring addictions, adjusted for sociodemographic variables (Model 1, 2) and other addictions (Model 2; n = 488).

		Bipolar disorder			Social anxiety disorder		
AUD combined with addiction to…	n	%	Model 1 OR [95% CI]	Model 2 OR [95% CI]	%	Model 1 OR [95% CI]	Model 2 OR [95% CI]
Alcohol (total)	488	8.7%			28.8%		
Cannabis	103	17.9%	**2.54 [1.28, 5.05]**	1.99 [0.94, 4.23]	38.6%	**1.67 [1.04, 2.68]**	1.36 [0.81, 2.28]
Tobacco	135	14.3%	**2.30 [1.17, 4.52]**	1.94 [0.93, 4.03]	34.6%	1.49 [0.96, 2.32]	1.29 [0.80, 2.07]
Internet	54	22.2%	**3.13 [1.43, 6.84]**	**2.76 [1.12, 6.79]**	57.3%	**3.70 [2.05, 6.68]**	**2.49 [1.29, 4.81]**
Gaming	53	13.4%	1.72 [0.70, 4.24]	0.88 [0.32, 2.41]	51.1%	**3.06 [1.69, 5.54]**	**2.12 [1.12, 4.02]**
Smartphone	89	10.1%	1.30 [0.58, 2.89]	0.85 [0.34, 2.15]	38.3%	**1.78 [1.09, 2.90]**	1.15 [0.66, 2.03]
Internet sex	73	12.1%	1.44 [0.63, 3.30]	1.18 [0.47, 2.94]	41.5%	**1.87 [1.10, 3.16]**	1.59 [0.90, 2.81]
Gambling	23	17.2%	2.34 [0.72, 7.66]	1.63 [0.44, 6.04]	47.4%	**2.38 [1.00, 5.63]**	1.31 [0.51, 3.36]
Work	52	21.5%	**2.95 [1.33, 6.54]**	**2.62 [1.13, 6.08]**	46.7%	**2.22 [1.22, 4.04]**	**1.89 [1.00, 3.55]**
Exercise	11	0.6%	-	-	35.8%	1.51 [0.43, 5.30]	1.36 [0.36, 5.16]

Notes: AUD = alcohol use disorder. ORs in bold are significant at p < .05. CI = 95% confidence interval. Adjusted for age, linguistic region and highest level of education (Model 1, 2). Model 2 is additionally adjusted for all other addictions (e.g. the regression for cannabis is adjusted by the eight addictions other than AUD and cannabis).

## 4. Discussion

Overall, our results were in line with the literature reporting strong associations between AUD and MHPs, especially in young people [6]. It has also been documented that co-occurring AUD and tobacco use disorder [22] as well as polysubstance dependence [15], respectively polysubstance use [13, 16–18, 20, 21], are associated with increased proportions of MHPs. The present study extends these findings to a number of other addictions, in that different SUDs and BAs co-occurring with AUD are associated with increased proportions of MHPs. Among participants with AUD plus at least one co-occurring addiction, the proportions of ADHD and social anxiety disorder were more than doubled and the proportions of MD and bipolar disorder were more than quadruple compared to participants with AUD alone (Table 3, step 2b). Thus, associations between AUD and MHPs were mainly due to the high proportions of MHPs in participants with AUD plus at least one co-occurring addictions.

However, our results also highlight the importance of comparing participants with AUD against the appropriate reference group: ORs for MHPs were, in general, only slightly above 1 and non-significant (and even below 1 for MD), if participants with AUD alone were compared to participants without AUD, but possibly with other addictions (Table 3, step 2a). On the other hand, if participants with no addiction at all (i.e. with none of our ten measured addictions) were taken as the reference group, ORs for MHPs in participants with AUD alone were considerably larger (all significant, except for MD; Table 3, step 3).

Overall, comparing the proportions of MHPs in participants with and without AUD can be severely biased if other co-occurring addictions are not taken into account. This seems especially true for MD, where the *unbiased* association (participants with AUD alone, compared to a reference group with no addiction) was substantially lower (Table 3, step 3; OR = 1.71 [0.85, 3.45]) than with the *standard* comparison between participants with and without AUD (Table 3, step 1; OR = 3.51 [2.73, 4.52]).

Regarding associations for specific combinations of addictions, the combination of AUD with all the other SUDs and BAs studied was associated with higher proportions of MHPs (except gambling for ADHD and exercise for bipolar disorder). These higher rates were not always significant, however, which may be due to the relatively small number of participants with this specific combinations of addictions, but also due to confounding with the effect of AUD on MHPs. Additionally, these associations were considerably reduced, often to non-significance, when adjusted for the presence of all other addictions. This indicates that at least part of the association between an addiction co-occurring with AUD and MHPs was not only due to the co-occurrence of that specific addiction with AUD, but may also have been shared with the association of a third (or additional) addiction with MHPs. It thus appears that the co-occurrence of addictions with AUD increased the odds of having MHPs in a rather general way. Additionally, results showed that the proportions of MHPs were also increased among participants with addictions other than AUD, especially if more than one addiction was present. This indicates that the results presented here may not be limited to AUD, but that the (co-)occurrence of other addictions is also associated with increased proportions of MHPs.

There are several possible explanations for the links between AUD, co-occurring addictions and MHPs ([53, 54] for an overview): a) AUD in conjunction with other co-occurring addictions leads to MHPs, or b) MHPs lead to AUD and often to other co-occurring addictions; c) alcohol and other addictive substances or behaviours are used as coping strategies for MHPs, or d) symptoms of MHPs are induced by alcohol use and other addictions, or e) the symptoms of MHPs, AUD and co-occurring addictions are caused by the same underlying vulnerability; and finally f) co-occurrence may be explained by similarities in the questionnaires assessing MHPs and addiction. Although our cross-sectional results cannot provide information about the causality of the associations observed, it is likely that all the pathways mentioned above are involved to some degree, and the links between AUD, co-occurring addictions and MHPs may be quite heterogeneous [53]. For MD and AUD, there is some evidence for a causal link in both directions [7], whereas for the other MHPs investigated there is no conclusive evidence that AUD or SUDs in general cause the onset of these conditions. ADHD, in particular, is considered to be an early onset disorder strongly influenced by genetic risk and family history [55], and therefore an explanation of ADHD as a risk factor for AUD and co-occurring addictions may be favoured. However, there remains the possibility that existing, but perhaps subthreshold ADHD symptoms were worsened because of the presence of addictive disorders.

Overall, our results suggest that a broad range of co-occurring addictions should be considered when studying the links between AUD and MHPs. Studies that do not consider co-occurring SUDs and BAs may be considerably biased or at least omit a potentially important covariate. Our results may also have implications for the treatment of patients with AUD. For example, co-occurring tobacco use disorder may not just increase the risk of diseases like lung cancer, but it may also be associated with co-occurring MHPs. According to findings from this study, the co-occurrence of AUD plus other addictions are strongly associated with a higher risk of MHPs. Therefore, when planning care, it would seem important that clinicians determine any co-occurrences of other addictive disorders with AUD, since they may be indicative of more complex clinical situations that are often associated with co-occurring MHPs. Earlier research pointed to the need of identifying optimal strategies for the care for patients with alcohol dependence who also use other substances [16]. The results of our study provide strong support for this and point additionally to the importance of also considering non-substance related addictive behaviours. The existing literature suggests that an integrated approach—one targeting all disorders simultaneously—is beneficial [6, 56, 57], but this is dependent on identifying all the various disorders [58]. Further investigations into the associations between SUDs, BAs and MHPs (including a broader range of MHPs potentially associated with addictions), together with improved, validated instruments, might help clinicians in their assessment of this complex issue.

### 4.1 Limitations

The present results must be seen in the light of the specificities of our sample, which was restricted to young Swiss men. Our findings therefore need replication among samples with a broader range of participants, especially including women and older people, and with a broader range of addictions and mental health problems. The present study used exclusively self-reported, relatively brief screening questionnaires with limited accuracy compared to clinical face-to-face evaluation and positive diagnoses may include rather mild cases without fully developed disorders. A replication of our results with professional clinical diagnoses would therefore be valuable. Longitudinal associations should also be tested, as will be done in the upcoming fourth wave of the C-SURF study. Of the seven behavioural addictions investigated in our study, only gambling disorder is currently recognised as a mental disorder in the DSM-5 [11], whereas there is an ongoing debate about some of the other behavioural addictions included [12, 59–61]. The instruments and the cut-offs used in this study, especially for BAs, still need more validation. Finally, to ensure comparability with other studies we used the time frames (for example in the last 6 or last 12 months) proposed by the authors of the original instruments and as a consequence, there are different time frames across scales in our study.

### 4.2 Conclusions

To the best of our knowledge, the present study is the first to demonstrate such a strong link between the co-occurrence of AUD and a broad range of other addictions and MHPs. The associations between AUD and MHPs are mainly due to the high proportions of MHPs among study participants with AUD plus at least one co-occurring addiction. Participants with AUD alone may even be no more likely to present with MD than participants without AUD. The study also showed that the link between AUD and MHPs may be biased, particularly for MD, if other addictions are not taken into account. The present study’s results have significant implications for research and treatment. Future research should consider the potential influences of other SUDs and especially also BAs when investigating the associations between AUD and MHPs. Regarding treatment, results suggest that clinicians should screen patients reporting AUD not only for other SUDs and MHPs, but also for BAs. Participants with multiple conditions may benefit from an integrated treatment approach [6, 56, 57], which targets AUD, other SUDs, BAs and MHPs simultaneously.

## Abbreviations

ADHD: Attention deficit hyperactivity disorder
AUD: Alcohol use disorder
BPD: Bipolar disorder
MD: Major depression
MHP: Mental health problems
